# MXene-Based Flexible Electrodes for Electrophysiological Monitoring

**DOI:** 10.3390/s24113260

**Published:** 2024-05-21

**Authors:** Meera Alex, Kashif Rast Baz Khan, Amani Al-Othman, Mohammad H. Al-Sayah, Hasan Al Nashash

**Affiliations:** 1 Biosciences and Bioengineering Graduate Program, American University of Sharjah, Sharjah P.O. Box 26666, United Arab Emirates; g00076143@aus.edu (M.A.); b00093614@aus.edu (K.R.B.K.); 2 Department of Chemical and Biological Engineering, American University of Sharjah, Sharjah P.O. Box 26666, United Arab Emirates; 3 Department of Biology, Chemistry and Environmental Sciences, American University of Sharjah, Sharjah P.O. Box 26666, United Arab Emirates; 4 Department of Electrical Engineering, American University of Sharjah, Sharjah P.O. Box 26666, United Arab Emirates; hnashsh@aus.edu

**Keywords:** MXene, bioelectrodes, flexible and wearable electronics, electrophysiology

## Abstract

The advancement of flexible electrodes triggered research on wearables and health monitoring applications. Metal-based bioelectrodes encounter low mechanical strength and skin discomfort at the electrode–skin interface. Thus, recent research has focused on the development of flexible surface electrodes with low electrochemical resistance and high conductivity. This study investigated the development of a novel, flexible, surface electrode based on a MXene/polydimethylsiloxane (PDMS)/glycerol composite. MXenes offer the benefit of featuring highly conductive transition metals with metallic properties, including a group of carbides, nitrides, and carbonitrides, while PDMS exhibits inherent biostability, flexibility, and biocompatibility. Among the various MXene-based electrode compositions prepared in this work, those composed of 15% and 20% MXene content were further evaluated for their potential in electrophysiological sensing applications. The samples underwent a range of characterization techniques, including electrochemical impedance spectroscopy (EIS), cyclic voltammetry (CV), as well as mechanical and bio-signal sensing from the skin. The experimental findings indicated that the compositions demonstrated favorable bulk impedances of 280 and 111 Ω, along with conductivities of 0.462 and 1.533 mS/cm, respectively. Additionally, they displayed promising electrochemical stability, featuring charge storage densities of 0.665 mC/cm^2^ and 1.99 mC/cm^2^, respectively. By conducting mechanical tests, Young’s moduli were determined to be 2.61 MPa and 2.18 MPa, respectively. The composite samples exhibited elongation of 139% and 144%, respectively. Thus, MXene-based bioelectrodes show promising potential for flexible and wearable electronics and bio-signal sensing applications.

## 1. Introduction

Bioelectrodes, also known as biological electrodes, serve as interfaces within the body to record physiological activity and transmit it to an instrumentation system [1]. Recent research focuses on the integration of bioelectrodes as a pivotal component in flexible electronics, which includes applications such as wearables, implantable electronics epidermal devices, health monitoring, stimulation systems, and human–machine interfaces [2,3]. Effortless monitoring of bioelectric signals such as electrocardiogram (ECG) [4,5], electroencephalogram (EEG) [6,7,8], and electromyogram (EMG) [9] necessitates the use of surface electrodes capable of signal capture from skin surfaces with minimal discomfort. Flexible noninvasive electrodes (FNEs) are becoming more and more popular around the world due to their close match to human tissues, which reduces pain and irritation during measurement [10,11].

The prevalent bioelectrodes found in the market are typically metal-based, posing significant challenges due to their inflexibility. In the design of surface electrodes, flexibility is a crucial consideration as it not only prevents damage to the skin and underlying tissues but also improves wearable comfort [2]. Proximity between the skin and electrode is essential for surface electrodes to uphold high signal-to-noise ratio (SNR) and accuracy, especially during movement [2].

Conventionally, Ag/AgCl electrodes are used for electrophysiological signal recording. These metal-based electrodes are extensively employed in health monitoring applications, primarily owing to their excellent electrical stability and economical production costs [12]. For effective electrical connectivity, traditional electrodes employed in electrophysiological studies depend on an electrolytic gel to establish a connection between the skin and a silver/silver chloride (Ag/AgCl) layer on the electrode surface [12]. However, the gel can irritate patients and dry up over time, reducing the strength of the signal and making it unpleasant to use over an extended period [13,14]. In addition to metal-based surface electrodes, researchers have explored various conductive polymers capable of achieving performance levels comparable to Ag/AgCl electrodes. One such example is the conformable and adhesive polymer electrode (CAPE) developed by Yang et al. [15]. Metal electrodes in the literature are functional in the scope of electrical conductivity but lack the necessary mechanical properties that are their ultimate downfall. In recent studies, the focus has turned toward incorporating highly conductive materials into conductive polymers and other polymers with notable biocompatibility and flexibility, yet limited conductivity. Among these polymers, polydimethylsiloxane (PDMS) has emerged as the most successful, demonstrating exceptional biocompatibility, flexibility, and in vivo stability [16]. Nevertheless, this polymer showed limited conductivity. To address this challenge, PDMS was combined with highly conductive materials, yielding a novel material that preserves the advantageous and conductive characteristics of the added material. A study by Alatoom et al. [17] developed nanosized titanium dioxide/PDMS electrodes that had better results than the previous study with excellent electrochemical properties as well as elasticity that was very close to biological tissue. Both these studies utilized metal fillers, but other studies explored the possibility of compositing less harsh conductive polymers with PDMS. In a recent study, Ali et al. created flexible PDMS composite electrodes featuring boronic acid-modified carbon dots for the recording of surface electrophysiological signals [18]. These electrodes exhibited a signal-to-noise ratio (SNR) of 36.75 dB, which is comparable to commercially available Ag/AgCl electrodes, along with a conductivity of 9.62 × 10^−3^ S/cm. MXenes have generated significant attention in biomedical research due to their ultrathin structure and intriguing properties, including electronic, optical, magnetic, and others [19,20,21]. Belonging to the category of 2D nanomaterials, MXenes consist of early transition metal carbides, carbonitrides, and nitrides, showcasing promising attributes such as high electrical conductivity, strength, flexibility, and volumetric capacitance [22,23]. This study proposes a combination of MXene and PDMS to utilize the attractive electrochemical properties of MXenes while retaining the biocompatibility and inherent flexibility of PDMS.

Flexible bioelectrodes present numerous advantages compared to traditional metal-based electrodes. Several materials were studied for self-powered applications [24,25]. While MXene composites have been extensively studied for biosensing and drug delivery applications [26,27], their exploration in the realm of surface electrodes is relatively nascent in biomaterial research. There is currently a scarcity of studies specifically investigating the use of MXene composites for biomedical signal acquisition purposes. To the best of the authors’ knowledge, studying a composite electrode based on the combination of MXene, PDMS, and glycerol has not yet been addressed in the literature. Furthermore, the evaluation of such composition electrochemically and mechanically has not yet been reported. Therefore, it is the objective of this work to describe such fabrication, evaluate various compositions, and provide electrochemical, surface morphology, and mechanical materials characterization. To address the challenges with the existing stiff bio-electrodes, this study aims to fabricate novel and flexible electrodes made from MXenes and PDMS polymers utilizing the electrochemical and mechanical properties of these two materials. This unique combination of MXenes, PDMS, and glycerol in electrode fabrication enhances electrode performance, functionality, and versatility, contributing to flexibility while providing a novel combination of materials and enabling the development of extremely flexible, advanced electrochemical devices for various wearable electronics applications. This electrode is hypothesized to have lower impedance, lower Young’s modulus, improved biocompatibility, and stability.

## 2. Materials and Methods

### 2.1. Preparation of Sample

The components employed for fabricating the bioelectrode consisted of Ti_3_C_2_ MXene powder purchased from Luoyang Advanced Material (Luoyang, China), polydimethylsiloxane (PDMS) formulated with the silicone elastomer base Sylcap TM 284-S (standard cure) and the silicone curing agent Sylcap TM 284-S (standard cure) both obtained from Microlubrol (Clifton, NJ, USA), along with glycerol (CAS# 56-81-5) sourced from Thermo Fisher Scientific (Waltham, MA, USA). To prepare the MXene-based electrode, a PDMS elastomer was added to a curing agent (10:1 proportion) at room temperature. MXene powder and glycerol were then added and thoroughly blended to create a homogeneous mixture. MXenes materials were used as received. No signs of agglomeration were observed in the samples. The composite mixture was well blended for at least 5–10 min depending on the samples’ size until a homogenous mixture was obtained. Glycerol, a well-dispersing agent, was used during the mixing process. Subsequently, the mixture underwent desiccation to eliminate air bubbles. The MXene/PDMS/glycerol blend was poured into a tailor-made tray with precise dimensions and left to cure in a fume hood for 24 h. Table 1 shows comparisons between various mass compositions of the prepared bioelectrode samples.

### 2.2. Sample Characterization

Sample characterization was performed to investigate which combination of the above-listed compositions provided the best performance while maintaining the structural integrity of the electrodes. This involved thorough ratio testing with various sample characterization techniques, as described below.

#### 2.2.1. Electrical Impedance Spectroscopy (EIS) Method

**Figure 1:**
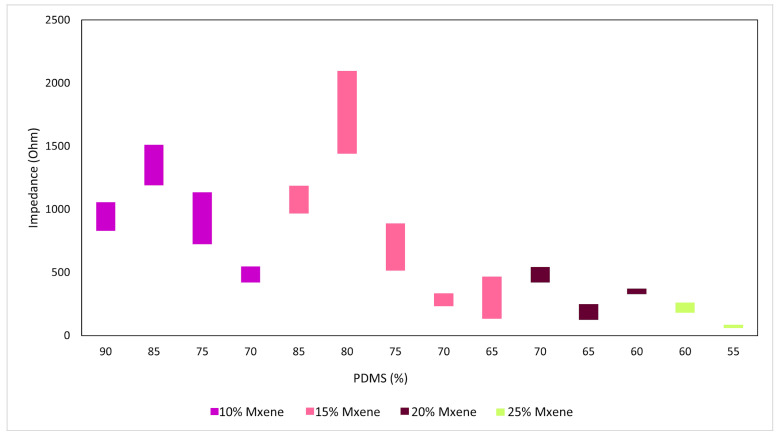
Range of bulk impedance.

The electrochemical impedance spectroscopy (EIS) technique is commonly employed to observe the response of a sample under the effects of an electric field where the frequency is either constant or changing. It is used to determine the properties of the coatings and soft materials. The sample underwent the application of an AC signal, with the resulting current and voltage measured to determine both real and imaginary impedances. The obtained data were plotted against each other over a range of frequencies. The electrochemical impedance spectroscopy (EIS) was conducted using a potentiostat (SP-200; Biologic, Seyssinet-Pariset, France). This potentiostat had a customized cell made up of two stainless steel electrodes with a cross-sectional area of 0.7854 cm^2^. The sample was securely clamped between the electrodes. A 10 mV voltage was administered, with the frequency spanning from 7 MHz to 100 MHz. The responses of the samples were recorded using the EC Lab software (v11.02). Resistance at a frequency of 1 kHz represents the neural activity frequency [28]. To determine the conductivity (σ) of the samples, Equation (1) was employed, incorporating values for the cross-sectional area (A), sample thickness (t), and resistance at the x-intercept (R). [29]. The samples’ resistance was also obtained from EC-lab fitting. The range of bulk resistance has been plotted in Figure 1.

(1) $$\sigma =\frac{t}{R\times A}$$

#### 2.2.2. Cyclic Voltammetry (CV) Method

Cyclic voltammetry (CV) is a widely used electrochemical technique to assess the stability of materials under certain operating voltage windows. CV is particularly effective in detecting and characterizing redox reactions at the electrode surface. If these reactions happen at any voltage, a peak will show in the CV graph. The absence of any peaks reveals that the sample is stable under these voltage conditions and does not undergo any oxidation/reduction reactions. Furthermore, the integral of the enclosed area in the CV graph is used to calculate how much the electrode surface can store charge. This is useful for neural stimulation applications. It involves applying a spectrum of potentials to the sample and recording the resulting current. The voltage and current data were then graphed as voltammograms.

The charge available at the electrode interface area (here it is called charge storage density (CSD) in C/cm^2^)) helps to determine the maximum amount of charge density allowed during the electrical stimulation process. This can be calculated by finding the integration of the cathodal area (the negative one) enclosed in the CV graph and then dividing it by the sweeping or scan rate in (mV/s) [30,31] and the electrode active area, as shown in Equation (2) below.

(2) $$\frac{Area\;below\;baseline}{Sweep\;or\;scan\;Rate\times electrode\;active\;area}$$

The CV measurements were conducted in solid-state mode. The sample was sandwiched between two stainless steel electrodes and connected to the potentiostat. The potentiostat (SP-200, Biologic) was used to perform the CV test using the same software (EC lab software v11.02) to record the results. The applied voltage was −1 V to 1 V.

#### 2.2.3. Mechanical Testing (Youngs Modulus)

To assess the mechanical characteristics of the samples, a quasi-static uniaxial tension test was conducted utilizing the Instron instrument (5582 Universal Testing Method, Instron, following the ASTM D638 standard). In this approach, a triplicate sample undergoes controlled tensile forces until the sample fails, typically resulting in breakage. The stress–strain data obtained during the test were recorded and plotted. From these data, Young’s modulus of elasticity, elongation percentage, and maximum stress before breakage were calculated as indicators of flexibility, durability, and ductility. Young’s modulus is the segment of the curve where stress is directly proportional to strain in the stress–strain graph. In the test, a sample undergoes controlled tensile force until failure. The data were then plotted in the stress–strain graph from which the modulus of elasticity can be observed. Furthermore, the samples were stretched at a rate of 5 mm/min until a sample failure was achieved. The samples in this work were 52 mm in length, 7 mm in width, 3 mm in height, and rectangular in shape. The linear region was established by plotting a linear trendline along the different regions of the graph and finding the best fit. The best linear fit was during the initial 0.03 to 0.05 strain values due to the samples being very soft.

#### 2.2.4. ECG and EMG Testing

To test the performance of the prepared electrodes in a real-time application, ECG and EMG testing were recorded using MXene/PDMS electrodes. For this experiment, customized MXene/PDMS electrodes were prepared using the casts described in Section 3.2, which incorporated the steel button from an Ag/AgCl electrode as a connection point, as shown in Figures 3–8. Without this steel button, it would have been difficult to obtain a secure connection to the recording apparatus. A Power Lab 26T Bio amplifier (AD Instruments, Sydney, Australia) was used to amplify the signals from the electrodes, and recording was performed using Lab Chart 7 software. Ag/AgCl electrodes, which are the standard electrodes used for these applications, were first used to record ECG and EMG signals as controls, and the signals recorded by the MXene/PDMS electrodes were compared.

## 3. Results and Discussion

### 3.1. Sample Composition Identification

**Figure 2:**
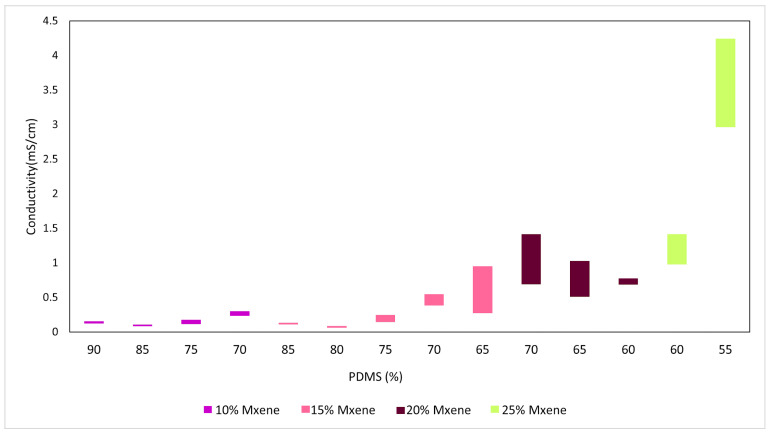
Range of conductivity.

An extensive batch of samples was prepared to determine which combination of MXene: PDMS: glycerol exhibited the most favorable initial electrochemical properties. The criterion was that the sample should be able to withstand repeated electrochemical testing in a customized potentiostat cell without physical degradation and exhibit approximately the same low bulk impedance and high conductivity for a triplicate sample. The bulk impedance (obtained from the Nyquist plot of each sample as well as the EC-lab software v11.02 fitting) and conductivity (calculated using Equation (1) of the various samples are shown in Figure 1 and Figure 2. The range of the preceding values is also reported in the figures. In this case, and due to the small sample size, the range was selected instead of the standard deviation for better representation of the data variation.

**Figure 3:**
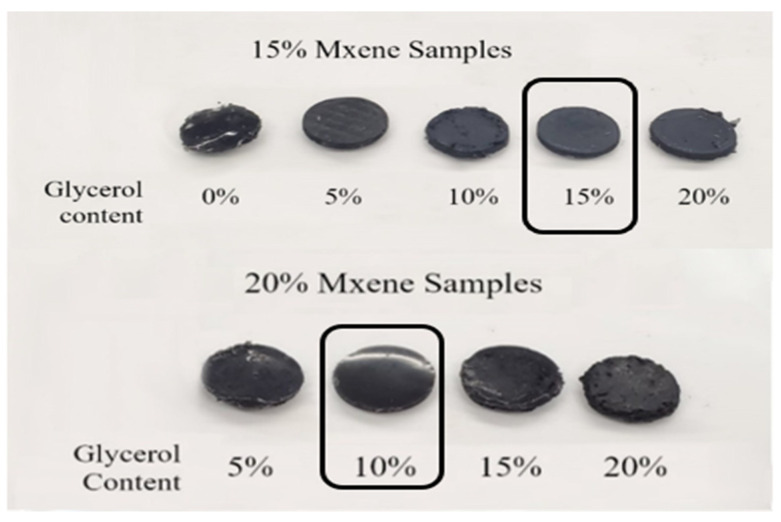
Pictures of samples after electrochemical testing was performed on each one. The highlighted sample compositions were chosen for further testing (Compositions 1 and 2, 15% and 20% MXenes, respectively).

These figures exhibit the range of bulk impedances and conductivity for various compositions. Conductivity values in the table show that, with increasing concentration of MXenes, the bulk impedance decreases, and the conductivity improves as a result. The tabulated results for the 10% and 15% MXene compositions show that increasing the composition of glycerol has the same effect. Figure 3 shows how the mechanical stability of each composition (upon visual inspection) is affected by stress, as they are tested in the potentiostat cell. The best performing compositions chosen based on the above criteria are as follows: (a) Composition 1:15% MXene: 70% PDMS: 15% glycerol, and (b) Composition 2: 20% MXene: 70% PDMS: 10% glycerol.

In summary, the electrochemical characterizations conducted on the prepared samples in this study indicate that the most suitable MXene mass compositions are 15% and 20%, respectively. It was observed that with increasing concentration of MXenes, the bulk impedance decreases, and the conductivity improves as a result. The bulk impedance of the MXene-based samples prepared in this study is significantly lower than that reported in previous studies that utilized PEDOT [32], PANI [33], and PMMA [31]. The following subsections include tests performed on the best-chosen compositions as described above. It should be noted that samples prepared with higher MXenes compositions appeared to be mechanically unstable. Please refer to Supplementary Information (Figure S1). This is another reason why Compositions 1 and 2 were selected for subsequent analyses.

### 3.2. Electrochemical Impedance Spectroscopy (EIS) Results

**Figure 4:**
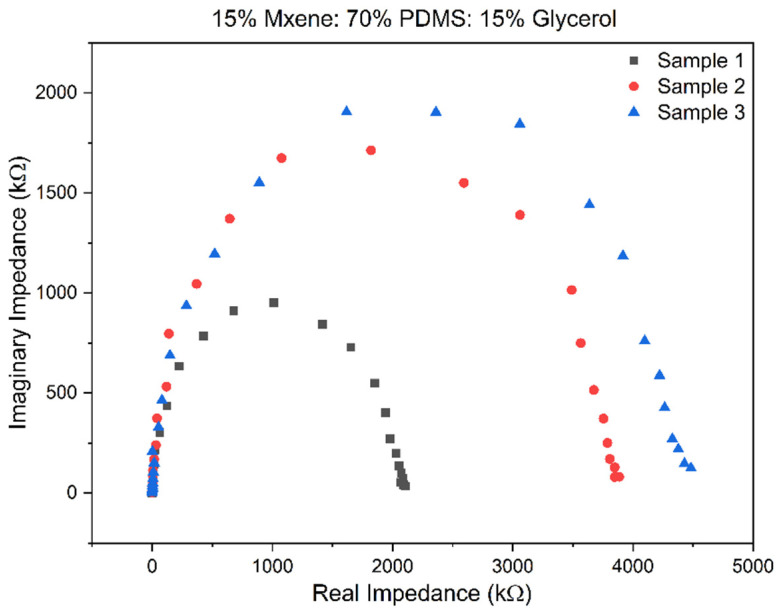
Nyquist plot for Composition 1.

**Figure 5:**
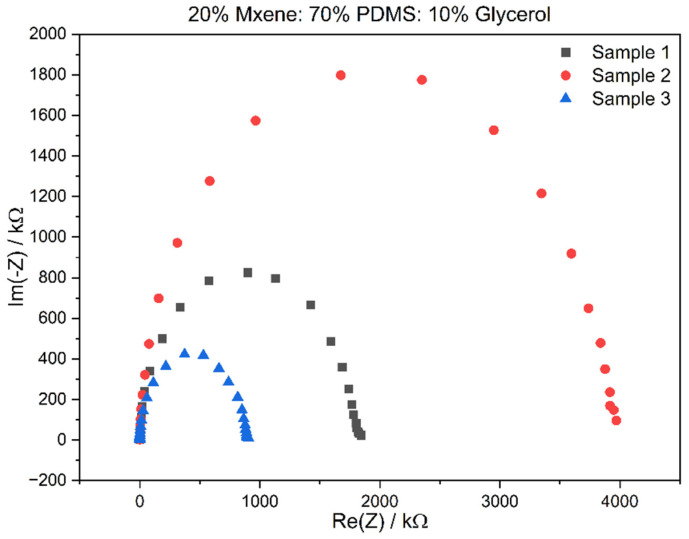
Nyquist plot for Composition 2.

Once the optimal compositions were chosen, EIS was performed on fresh samples of the before-mentioned compositions, and through this technique, the bulk impedance, impedance at 1 kHz, and conductivity were acquired to compare the two compositions and the literature. The Nyquist plot of the sample gives the real impedance at the x-intercept, which is known as the bulk impedance. Figure 4 and Figure 5 show the Nyquist plots for Composition 1 and Composition 2, performed in triplicate.

The bulk impedance for the triplicates of Composition 2 is in the range of 419–543 Ω, the 1 kHz impedance was 1.986 ± 1.566 MΩ, and the conductivity calculated was in the range of 0.688–1.424707 mS/cm. This was an improvement over Composition 1 as the impedances were lower, and the conductivity had improved. Table 2 in the following section compares the reported values to that of the literature. The prepared sample electrodes had a low bulk impedance in the range of 1.7–0.1 kΩ. The bulk impedance for the triplicates of Composition 1 is in the range of 233–334 Ω, the 1 kHz impedance was 1.986 ± 1.566 MΩ and the conductivity calculated was in the range of 0.455–0.381 mS/cm.

### 3.3. Cyclic Voltammetry (CV) Results

**Figure 6:**
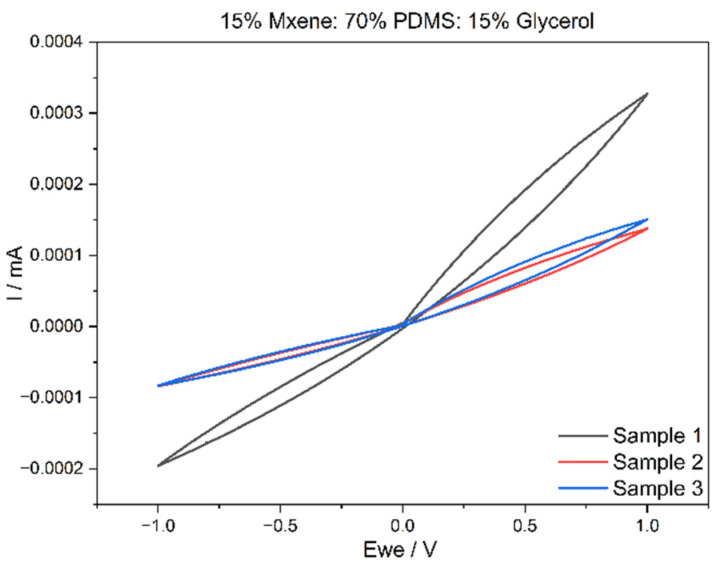
Composition 1 voltammogram.

**Figure 7:**
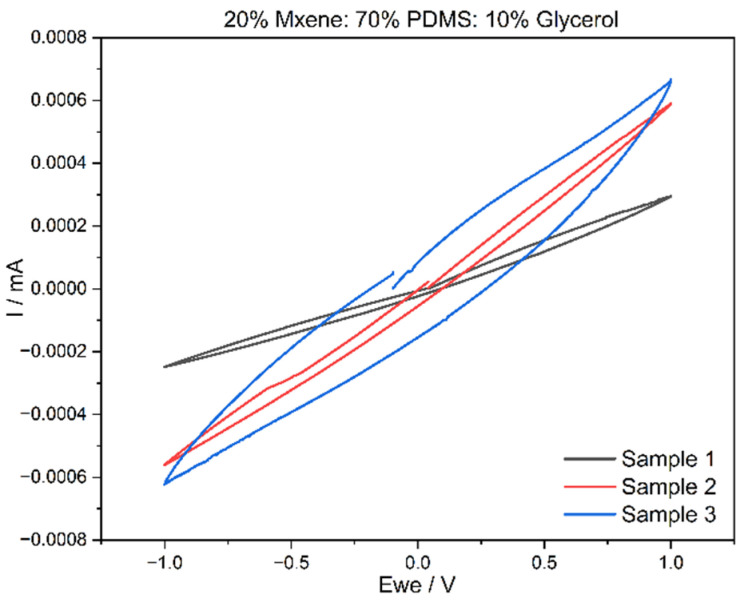
Composition 2 voltammogram.

Cyclic voltammetry was carried out on the two compositions from which the voltammograms, current against time graphs and, as a result, charge storage density (CSD) were recorded. Figure 4 and Figure 5 exhibit the voltammogram curves for Composition 1 and Composition 2 in triplicate. The CSD was determined for a scan rate of 20 mV/s with an electrode thickness of 1 mm. These parameters were kept constant for all CV testing completed through the experimental work. From the above graphs plotted in Figure 6 and Figure 7, both the compositions have very stable oxidation and reduction cycles with the absence of peaks. It can also be deduced that Composition 2 has higher current density values, which can be attributed to the higher MXene content.

For Composition 1, the CSD was 0.665 ± 0.33 mC/cm^2^ while the CSD for Composition 2 was 1.99 ± 1.25 mC/cm^2^. From these values, the increase in MXene content affects the CSD values positively. Achieving a higher CSD is not an objective for this material, as the purpose is to use it as a conductor with low impedance, rather than for current storage purposes. However, if the material was to be developed for energy storage purposes, then having a high CSD is vital. This can be completed by increasing the MXene content or reducing the surface area of the electrodes. Table 2 compares the CSD values to the literature. In addition to low bulk impedances and high conductivity, the MXene-based samples also possessed good charge storage density. In Composition 1 (15% MXene), the CSD was 0.665 ± 0.33 mC/cm^2^, while Composition 2 (20% MXene) exhibited a higher CSD of 1.99 ± 1.25 mC/cm^2^. These results indicate a positive correlation between the increase in MXene content and enhanced CSD values. It is important to note that achieving a higher CSD is not a primary objective for this material. The intended use is as a conductor with low impedance rather than for storing current. However, it is noteworthy that if the material were to be designed for energy storage purposes, a high CSD would be crucial. This can be accomplished by either increasing the MXene content or reducing the size of the surface area of the electrodes. These considerations underscore the versatility of the material, allowing it to be tailored for specific applications based on the desired electrical properties.

### 3.4. Mechanical Testing Results

**Figure 8:**
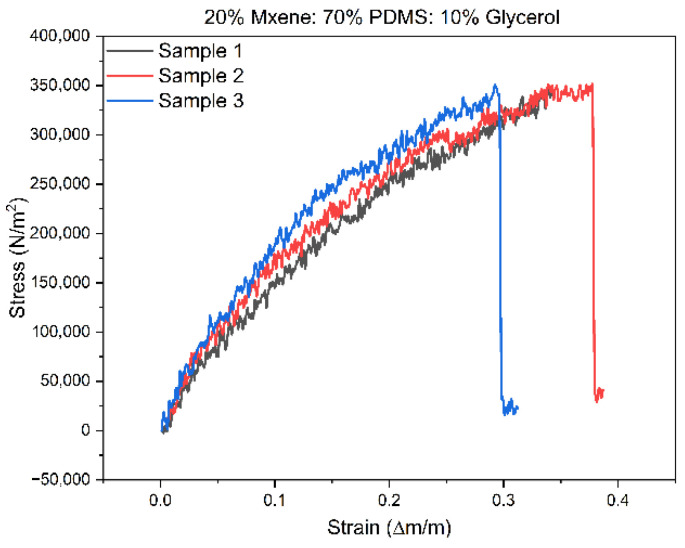
Stress–strain graphs of triplicate sample of Composition 2.

**Figure 9:**
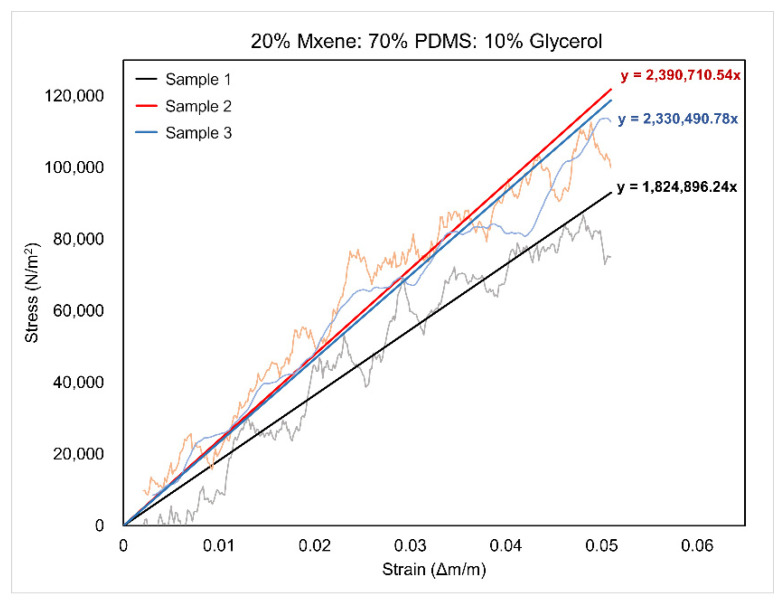
Trendlines for the elastic regions in the stress–strain graphs of Composition 2.

Three identical samples with dimensions of 52 mm in length, 7 mm in width, and 3 mm in height were fabricated for Compositions 1 and 2 to undergo mechanical testing. These samples were clamped in the tensile testing apparatus and stretched until failure in the form of breakage. The force applied and extension values were recorded, from which the stress–strain data were calculated and graphed. Figure 8 shows the stress–strain data for Composition 2. The average Young’s modulus was computed from the aforementioned graphs by determining the gradient of the elastic region, which corresponds to the linear portion starting from the origin, in the stress–strain graphs. This was completed by applying a trendline to those regions as shown in Figure 9. The results of the mechanical testing for Compositions 1 and 2 in comparison to other literature are shown in Table 3. Composition 2 has a lower Young’s modulus than Composition 1 and this was attributed to the increased content of the MXenes. Composition 2 also stretched by a higher percentage, meaning that the increase in MXene content made the final material more ductile. In general, for both compositions, these values are deemed satisfactory, considering that Young’s moduli fall within the range typical of skin properties (25 kPa–80 MPa under tensile and torsion tests) [35]. The prepared MXene-based samples demonstrated a notable level of flexibility comparable to that of skin tissue (25 kPa–80 MPa under tensile and torsion tests) [28,35].

### 3.5. ECG and EMG Testing

**Figure 10:**
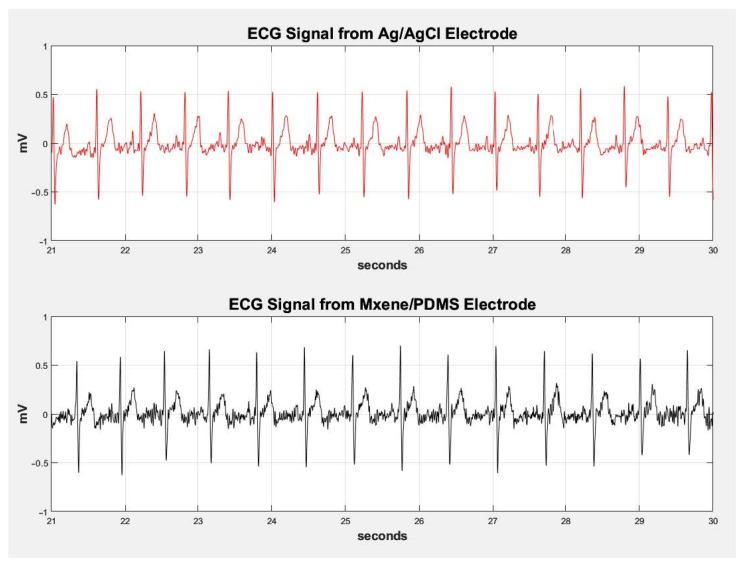
Ag/AgCl versus MXene/PDMS electrodes.

The ECG and EMG testing was performed to see how the electrode material compares to commercially available electrodes like Ag/AgCl. As such, the same electrode was used as a control for these experiments, to which the MXene electrodes were compared. The signals from both electrodes were plotted along a shared time axis in MatLab. Figure 10 shows the comparison of the ECG experiment between Ag/AgCl electrodes and MXene/PDMS electrodes. Please refer to the Supplementary Materials section for the setup details and sample preparation (Figure S6).

**Figure 11:**
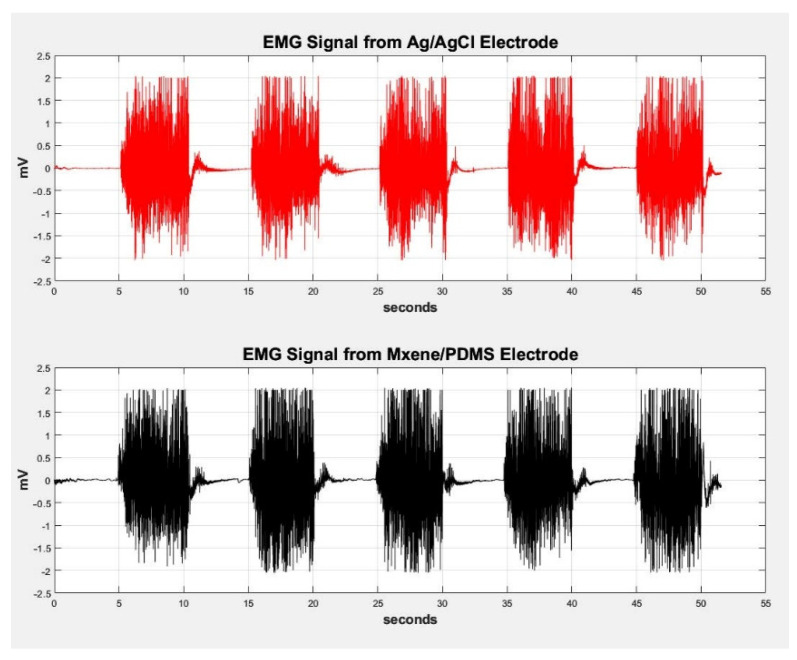
Comparison of EMG signal from the bicep muscle between Ag/AgCl and MXene/PDMS electrodes.

The PQRS regions are clearly visible in all segments of the ECG signals presented in the above figure for the MXene/PDMS electrode. The developed MXene/PDMS electrodes can detect biological signals from the skin with the same parameters used by commercial electrodes, however, there is slight noise in the MXene/PDMS electrode signal, but this can be attributed to not having enough gel at the skin–electrode interface. The MXene/PDMS electrode exhibits higher amplitude in certain peaks at seconds 26 and 27, suggesting potential increased conductivity compared to Ag/AgCl electrodes. Figure 10 illustrates the comparison of EMG signals between the Ag/AgCl electrodes and the MXene/PDMS electrodes. Figure 11 shows the contraction and relaxation of the bicep muscle in 5 s intervals. The contraction periods are aligned against a 50 s window for the signals of both electrode materials. The synthesized sample has excellent electrochemical and mechanical properties that make it best suited to flexible and wearable electronics sensing applications. The results of ECG and EMG recording prove that it can be concluded that the developed MXene/PDMS-based electrodes can also function as wearable flexible conductive electrodes for biosignal sensing on the skin.

## 4. Conclusions

Through this study, a novel bioelectrode based on Ti_3_C_2_ MXene/PDMS composite was developed for biosignal sensing purposes. The bioelectrode material, prepared and investigated in two compositions, had a multitude of electrochemical, mechanical, long-term immersion, and biological tests performed on it. The experimentation began with finding the best combination of MXene: PDMS: glycerol by performing a thorough ratio optimization test in which two compositions were chosen based on preliminary electrochemical testing. Composition 1 consisted of 15% MXene: 70% PDMS: 15% glycerol and Composition 2 consisted of 20% MXene: 70% PDMS: 10% glycerol. The bulk impedance for these compositions were 280 ± 41 Ω and 111 ± 78 Ω, respectively. The conductivities of Compositions 1 and 2 were 0.462 ± 0.07 mS/cm and 1.533 ± 0.88 mS/cm, respectively. The CV voltammograms showed stable oxidation/reduction curves without the presence of peaks and the charge storage density was calculated to be 0.665 ± 0.33 mC/cm^2^ and 1.99 ± 1.25 mC/cm^2^, respectively. Following electrochemical testing, mechanical characterization was conducted, from which the Young’s moduli of the compositions were determined to be 2.61 ± 0.39 MPa and 2.18 ± 0.31 MPa, along with elongations of 139 ± 11% and 144 ± 13%, respectively. These values are satisfactory as they fall within the mechanical range acceptable for skin. ECG and EMG testing showed that the developed material had excellent signal resolution and conductivity when compared to Ag/AgCl electrodes in both tests.

As the material performed exceptionally well in flexibility and in detecting biosignals from the skin, future work should be completed on developing the MXene/PDMS biomaterial for wearable skin sensors and electronics as the results were significant through ECG and EMG testing. Furthermore, the current samples offer very promising potential for long-term implantation. Overall, the MXene/PDMS composite developed through this thesis holds promising potential for further investigation.

## Figures and Tables

**Table 1:** Compositions prepared for optimization studies.

MXene (%)	PDMS (Elastomer + Curing Agent 10:1 Ratio Mixed Separately) (%)	Glycerol (%)
10	90	0
	85	5
	80	10
	75	15
	70	20
15	85	0
	80	5
	75	10
	70	15
	65	20
20	75	5
	70	10
	65	15
	60	20
25	60	15
	55	20

**Table 2:** Comparative list of bulk impedance, impedance at 1 kHz, conductivity, and CSD in relation to the literature.

Material	Bulk Impedance in kilo Ohms	Impedance at 1 kilo Hertz	Conductivity in mS/cm	CSD in mC/cm^2^
Polyamide/Cr-Ag-Cr [34]	22.7	120	-	-
PSS/PEDOT [32]	2.23	2.54	0.012	4.86 ± 0.24
PANI/PDMS [33]	0.6–4	1.6–28	10^−5^–0.01	-
TiO_2_/PDMS [17]	0.790	24	0.722	27 ± 1.1
TiO_2_/PMMA/Silicon [31]	4.28	114.6	0.1	1.23
PDMS/BA-CD/Glycerol [18]	0.058	964	9.62	0.0214
Composition 1 [this work]	0.280 ± 0.04	3192 ± 1041	0.462 ± 0.07	0.665 ± 0.33
Composition 2 [this work]	0.111 ± 0.07	1986 ± 1566	1.533 ± 0.88	1.99 ± 1.25

**Table 3:** Comparison to the literature of elongation and Young’s modulus values.

Material	Elongation	Young’s Modulus
PANI/PDMS based electrodes [33]	308%	75.312 MPa
TiO_2_/PDMS based implantable electrode [17]	293%	32.9 kPa
Silk fibroin/Ti_3_C_2_ film-based sensor for wearable pressure and electrical signal sensing [36]	—	1.22 MPa
Self-healing MXene/PDMS wearable biosensor [37]	181%	—
Composition 1 [this work]	139 ± 11%	2.61 ± 0.39 MPa
Composition 2 [this work]	144 ± 13%	2.18 ± 0.31 MPa

## Data Availability

Data are contained within the article.

## Supplementary Materials

The following supporting information can be downloaded at: https://www.mdpi.com/article/10.3390/s24113260/s1, Table S1: Ratio optimisation bulk impedances and conductivity with s.d. of triplicate samples. Figure S1: Pictures of samples after electrochemical testing was done on each once. The highlighted sample compositions were chosen for further testing. Figure S2: SEM images of Composition 1 (the 15% MXene-). Figure S3: Electrode cast with steel button for ECG and EMG testing and the prepared electrodes ready for testing. Figure S4: MXene 15% /PDMS Glycerol (Light microscope image). Figure S5: MXene 20% /PDMS Glycerol (Light microscope image). Figure S6: (a) Electrode sample used for ECG recorder fitted to an electrode lead (b) Power Lab Amplifier for ECG Data Recording (c) Mxene sample, (d) ECG Recording Setup.
