# Supplementary material for: MXene-Based Flexible Electrodes for Electrophysiological Monitoring
Source: Sensors (Basel). 2024 May 21;24(11):3260. doi: 10.3390/s24113260 (PMC11174813; doi:10.3390/s24113260)
Supplement: Supplementary file 1 [file sensors-24-03260-s001.zip › sensors-2877109-supplementary.pdf]

Supplementary information

Table S-1: Ratio optimisation bulk impedances and conductivity with s.d. of triplicate samples.

Mxene (%)	PDMS (%)	Glycerol (%)	Average Bulk impedance (Ω)	Average Conductivity (mS/cm)
10	90	0	913 ± 125.19	0.14 ± 0.017
	85	5	1314.37 ± 172.86	0.098 ± 0.012
	80	10	-	-
	75	15	914.41 ± 208.32	0.144 ± 0.032
	70	20	491.52 ± 65.22	0.262 ± 0.036
15 (composition 1)	85	0	1077.28 ± 157.37	0.119 ± 0.017
	80	5	1726.56 ± 335.24	0.075 ± 0.013
	75	10	700.47 ± 186.68	0.19 ± 0.052
	**70**	**15**	**285.66 ± 50.63**	**0.455 ± 0.083**
	65	20	256.43 ± 183.13	0.659 ± 0.34
20 (Composition 2)	80	0	-	-
	75	5	805.91 ± 782.79	0.271 ± 0.181
	**70**	**10**	**131.66 ± 48.56**	**1.054 ± 0.363**
	65	15	191.66 ± 63.51	0.723 ± 0.27
	60	20	350 ± 31.11	0.73 ± 0.064
25	75	0	-	-
	70	5	-	-
	65	10	-	-
	60	15	220.5 ± 57.27	1.195 ± 0.31
	55	20	73 ± 18.38	3.602 ± 0.907

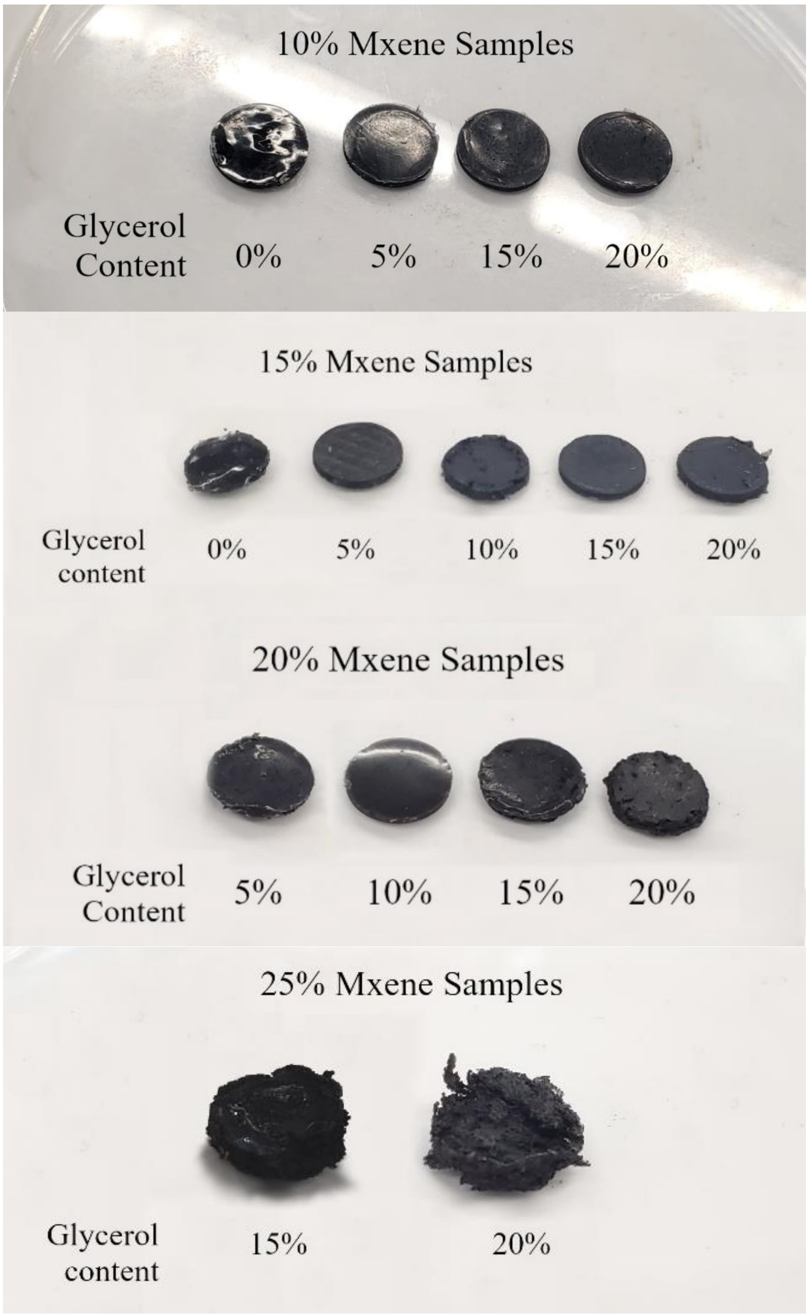

Figure S-1: Pictures of samples after electrochemical testing was done on each once. The highlighted sample compositions were chosen for further testing.

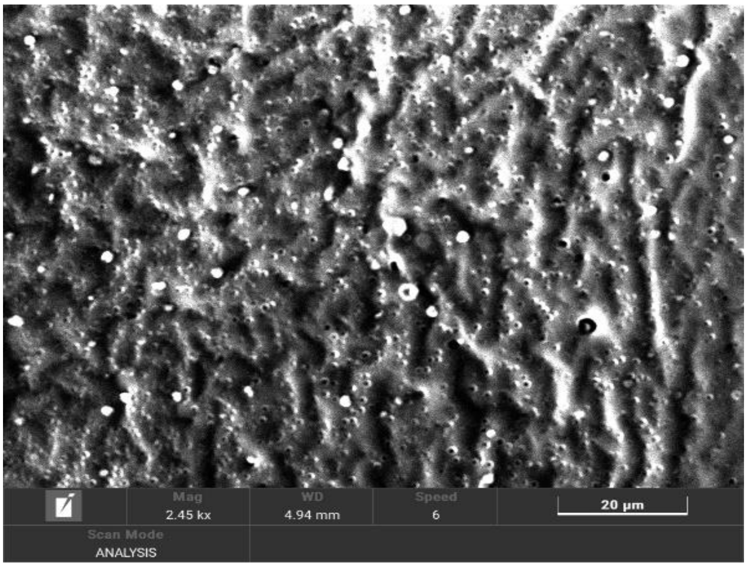

Figure S-2: SEM images of Composition 1 (the 15% MXene-)

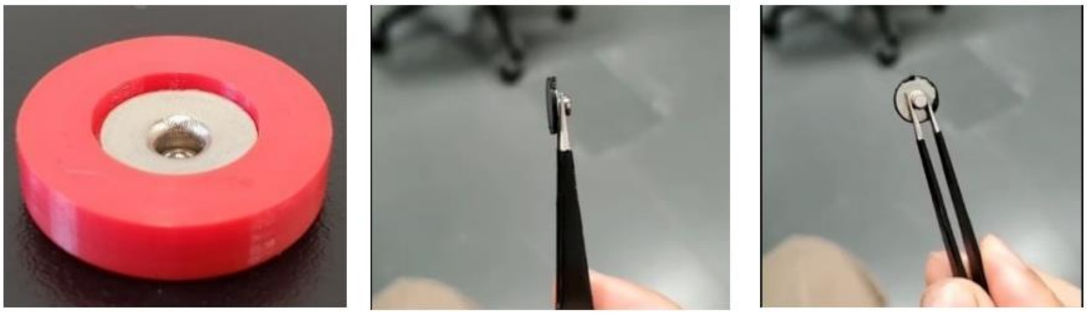

Figure S-3: Electrode cast with steel button for ECG and EMG testing and the prepared electrodes ready for testing.

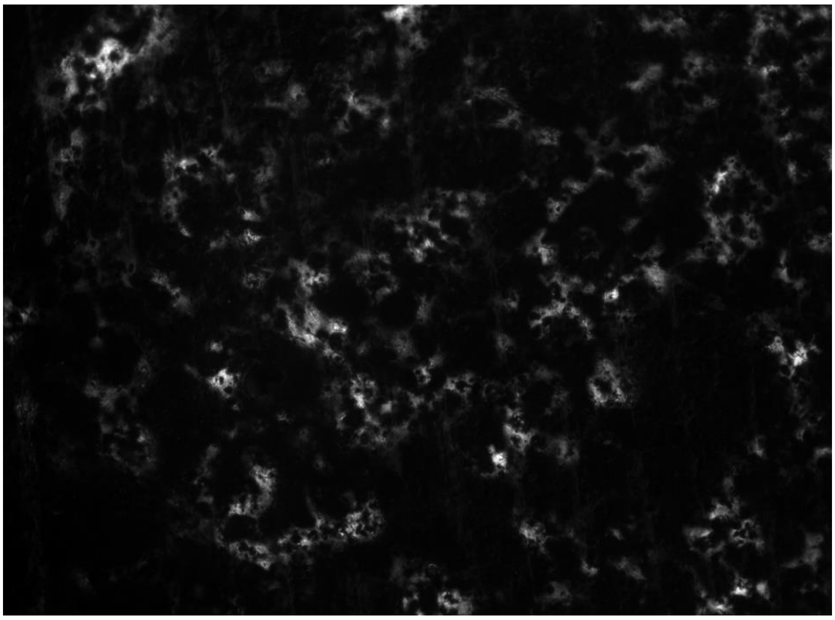

Figure S-4: MXene 15% /PDMS Glycerol (Light microscope image)

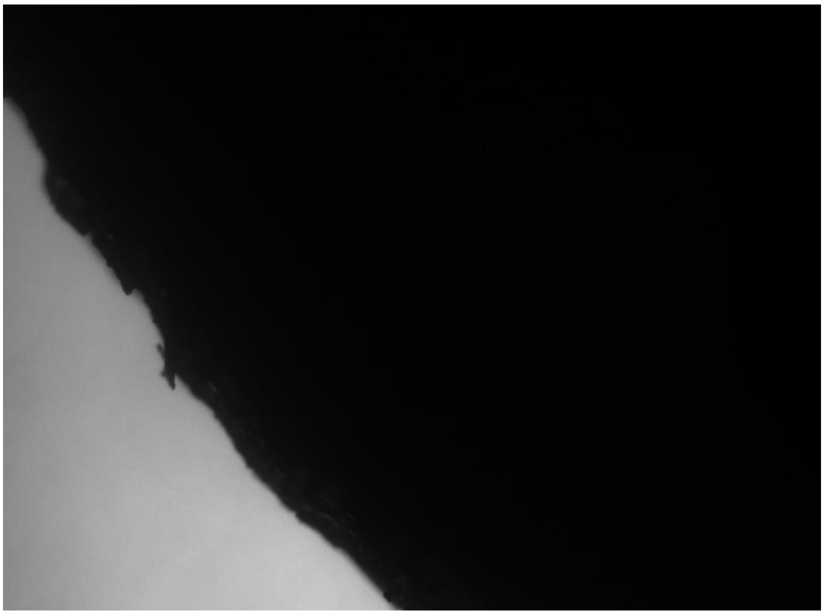

Figure S-5: MXene 20% /PDMS Glycerol (Light microscope image)

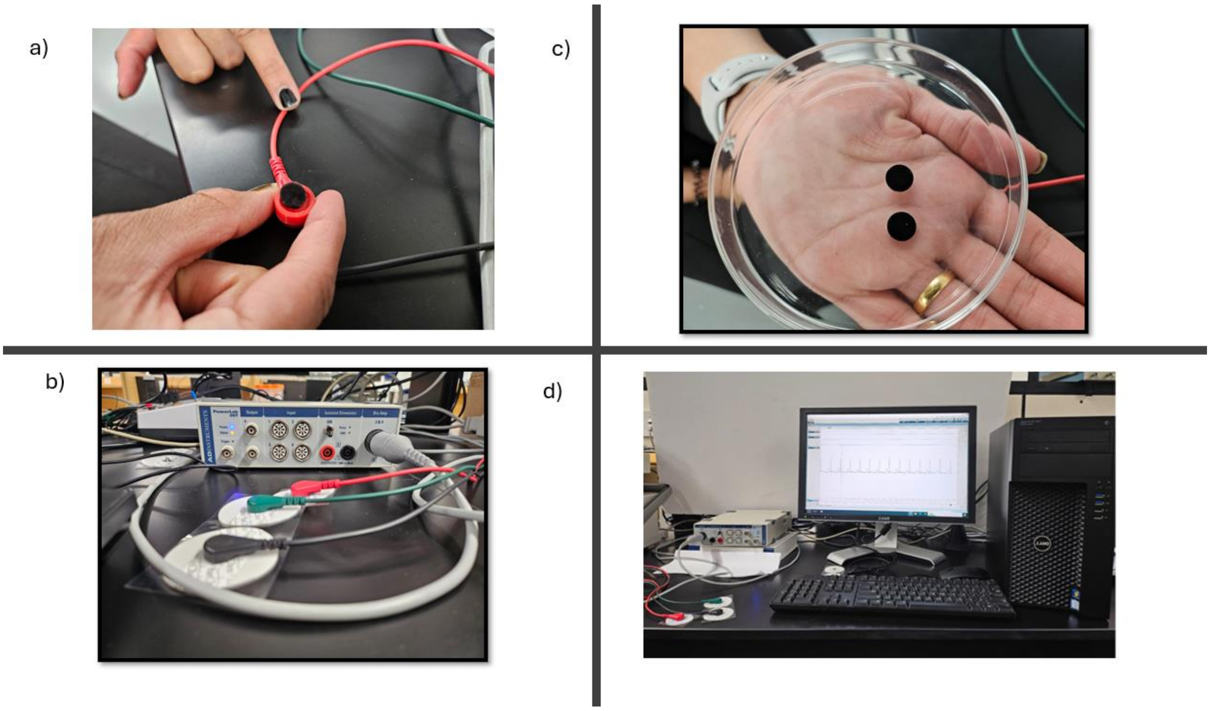

Figure S-6: (a) Electrode sample used for ECG recorder fitted to an electrode lead (b) Power Lab Amplifier for ECG Data Recording (c) Mxene sample, (d) ECG Recording Setup
